# Structural Characterization and Antioxidant Capacity of Quinoa Cultivars Using Techniques of FT-MIR and UHPLC/ESI-Orbitrap MS Spectroscopy

**DOI:** 10.3390/plants10102159

**Published:** 2021-10-12

**Authors:** Miguel García-Parra, Diego Roa-Acosta, Víctor García-Londoño, Brigitte Moreno-Medina, Jesús Bravo-Gomez

**Affiliations:** 1 Doctoral Program in Agriculture and Agroindustrial Science, Agriculture Department, Universidad del Cauca, Popayán 190002, Colombia; 2 Agroindustry Department, Faculty of Agricultural Sciences, Universidad del Cauca, Popayán 190002, Colombia; droa@unicauca.edu.co (D.R.-A.); jebravo@unicauca.edu.co (J.B.-G.); 3 Institute of Polymer and Nanotechnology, Facultad de Arquitectura Diseño y Urbanismo, University of Buenos Aires-CONICET, Intendente Güiraldes 2160, Buenos Aires C1428EGA, Argentina; vgarcia@qo.fcen.uba.ar; 4 Facultad de Ciencias Agropecuarias, Universidad Pedagógica y Tecnológica de Colombia (UPTC), Tunja 150002, Colombia; brigitte.moreno@uptc.edu.co

**Keywords:** Amaranthaceae, chromatography, polyphenol, quinoa starch, secondary protein

## Abstract

The existence of more of 16,000 varieties of quinoa accessions around the world has caused a disregard on their structural and phytochemical characteristics. Most of such accessions belong to cultivars settled in Colombia. The goal of this research was to evaluate the structural attributes and antioxidant capacities from six quinoa cultivars with high productive potential from central regions in Colombia. This study used middle-range infrared spectroscopy (IR-MIR) to determine the proteins, starch and lipids distinctive to quinoa grains. Ultra-high-performance liquid chromatography electrospray ionization Orbitrap, along with high-resolution mass spectrometry (UHPLC/ESI-Orbitrap MS), were also used to identify the existence of polyphenols in cultivars. The antioxidant capacity was determined through DPPH, ABTS and FRAP. The spectrums exhibited significant variances on the transmittance bands associated with 2922 cm^−1^, 1016 cm^−1^ and 1633 cm^−1^. Moreover, the intensity variations on the peaks from the secondary protein structure were identified, mainly on the bands associated with β-Sheet-1 and -2, random coil α elice and β-turns-2 and -3. Changes found in the ratios 996 cm^−1^/1014 cm^−1^ and 1041 cm^−1^/1014 cm^−1^ were associated with the crystalline/amorphous affinity. Regarding the antioxidant capacity, great differences were identified (*p* < 0.001) mainly through FRAP methods, while the phenolic acids and flavonoids were determined by UHPLC/ESI-Orbitrap MS techniques. The presence of apigenin and pinocembrin on grains was reported for the first time. Titicaca and Nariño were the most phytochemically diverse quinoa seeds.

## 1. Introduction

According to different scientific reports, quinoa had its origins in South America between Colombia and Chile. However, its greater abundance in terms of morphologic, ecophysiology and nutritional diversity happens in countries like Ecuador, Bolivia, Perú and Chile. The distribution of cultivars happens according to their source of origin as quinoa from: the altiplano Inter-Andean Valley, from the coast and from Salares and Yungas [1]. Quinoa species show a great adaptability to harsh edaphoclimatic conditions, mainly related to dramatic changes in the temperature, salinity stress and lower availability of water and nutrients [2].

Along with quinoa, amaranth, buckwheat and chia were underutilized for the effect of introduction in crops such as rice, wheat, soybean and barley. However, underutilized species are important components of the local agriculture and comprise a broad variety of crops that are traditionally used and that may have potential for adaptation to climate change, medicinal properties and functional food development [3]. However, the quinoa cultivars incorporated into genetic improvement can reduce the richness of this species [4,5].

Under tropical conditions, grains were the main nutritional source for many pre-Hispanic cultures. Nowadays, quinoa is recognized in the food industry for some of its nutritional and techno-functional properties linked to its lipidic, protein and starchy nature [6,7].

Since 2013, the FAO boost production, transformation and commercialization of quinoa crops has caused an exponential rise in grain consumption, as well as its byproducts. Such a fact has benefited the increase of crops areas to select those with better agroclimatic adaptability, therefore improving hectare production [3,8], while the high genetic diversity could allow identifying the most suitable cultivar for each agroecological condition, considering its productive and nutritional performance [1].

Furthermore, it is relevant to consider the influence of different external conditions of biotic, abiotic and genetic attributes of such cultivars in relation to the nutritional characteristics of grains to which some researchers reported their findings. Authors like Reguera et al. (2018) [9] explained the results under torrid and outer equatorial conditions. García-Parra et al. (2019) [10] addressed the outcome of using different fertilization levels and sources. Lesjak & Calderini (2017) [11] reported modifying nighttime temperatures. Miranda et al. (2013) [12] evaluated environmental conditions over two consecutive years. These findings about analyses related to the techno-functional characteristics of quinoa grains should be applied to all cultivars according to the agroclimatic conditions.

Agro-industrial and bioactive characteristics in Colombian quinoa cultivars have been understudied; they lack information or show discrepancies between cultivars [13,14]. According to previously described information, all properties must be taken into account at the time to evaluate the nature of quinoa for its use in the food industry.

The increase in demand means that the prices of quinoa seeds and flours has increased rapidly worldwide, also increasing the risk of possible adulterations, requiring rapid and efficient analytical techniques for this detection. Additionally, the use of quinoa to produce nutraceuticals and functional foods requires a rapid and efficient analysis of their characterizations and even the agricultural production in areas of appropriate agroecological conditions [15].

In parallel, some research had made progress by studying the agro-industrial quinoa’s precursor germplasm, as described by Rodríguez et al. (2019) [16]. They handled short and mid-range infrared spectroscopy to single out carbohydrates, lipids and proteins present from quinoa belonging to five different geographic areas. There is also an interest that has become relevant over the recent years around examining bioactive compounds and the antioxidant capacity of seeds [17,18].

Techniques such as mid-range spectroscopy (400–4000 cm^−1^) allow to pinpoint protein and starch secondary structures present in different quinoa cultivars. Along with other procedures to determine the phenolic compounds and antioxidant capacity, this practice grants a better understanding to determine the grain potential towards functional food. Therefore, this research hypothesis brings up a significant variation between the spectroscopy characteristics and bioactive compounds from different quinoa cultivars.

## 2. Results

### 2.1. Quinoa Grain Characteristics

**Figure 1:**
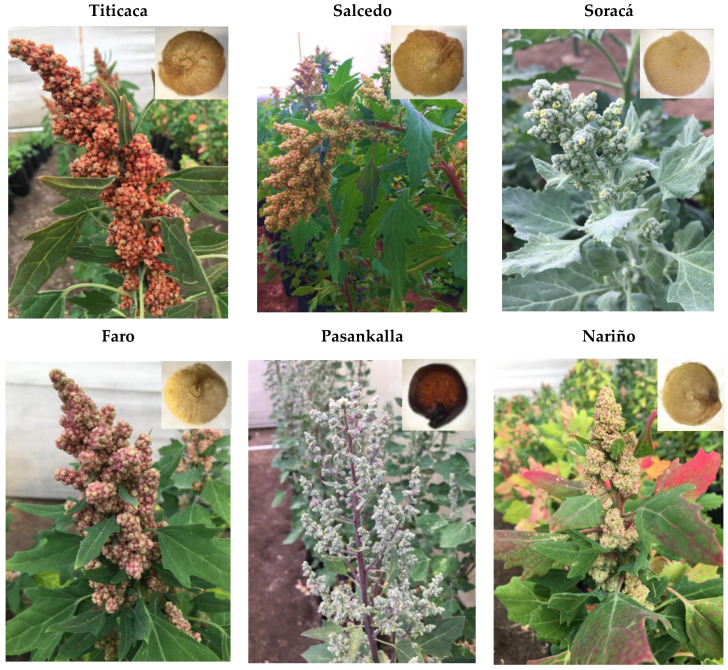
Macroscopic grains image from different cultivars in Colombia.

Color has recently become a classification factor for quinoa grains, as well as a quality attribute (Figure 1). Colombian cultivars show a cream-white color associated with genomic quinoas from the inter-Andean Valley region, with typical short days with a high sensitivity to luminosity [8,19]. However, the genetic diversity and increment of its consumption has brought an ease to its spreading to new cultivars, showing attributes linked to pericarpic coloring, dimensions and constitutive molecules.

The evaluated cultivar grains showed significant differences between the L*a*b* coordinates linked to external integument coloration. Such a condition happened to all the cultivars except Titicaca and Puno (on L*) and Salcedo and Puno on a*. The proteins, carbohydrates and fats were studied on all the cultivars. Titicaca and Pasankalla had greater protein values; Puno and Nariño were rich in carbohydrates, while the fats were greater in Puno region cultivars (Table 1).

### 2.2. Infrared Spectroscopy

The results allowed to identify a 1633-cm^−1^ transmittance linked to C=O stretching, which is also related to its protein composition [20]. Table 2 shows meaningful differences of the transmittance values from the cultivars. Since there is a direct relationship between the transmittance and protein, it is possible to say that Pasankalla is the one with a lesser protein value in contrast to Nariño, Salcedo and Puno, with greater amounts.

This way, band differences associated with C-H stretching and C-O-C and C-O folding were observed. About starch, a major proportion was observed on the Titicaca and Puno cultivars, while, in Soracá and Pasankalla, the rates were lower. The stretching of the C-H path group linked to lipids made it possible to identify the major differences among the cultivars.

#### 2.2.1. Secondary Protein Structure by FTIR Spectroscopy

Table 3 shows the bands after performing a deconvolution on amide I in the region between 1600 and 1700 cm^−1^. This is the most spectral-sensitive area to secondary protein structures but also responsible for the vibration and strain of the peptic C=O bond [21]. The amide I band spectrum allowed to determine the secondary structure protein for all six cultivar quinoa grains, as in the previously described parameter analysis protocols.

#### 2.2.2. Secondary Starch Structure by FTIR Spectroscopy

There were noticeable peaks observed on 800 cm^−1^ and 1300 cm^−1^ attributed to starch and corresponding to a flexing COH vibration. According to the data, the evaluated cultivars showed distinguished results (*p* < 0.05) on each region, the most intense at 996 cm^−1^ and 1076 cm^−1^. As for the 1041-cm^−1^ crystalline structure band, it had a greater intensity in Pasankalla and Nariño, while the 1014-cm^−1^ region had more shapeless structural intensity in Nariño (Table 4).

### 2.3. Polyphenols and Antioxidant Capacity by ABTS, DPPH and FRAP

Quinoa crops showed polyphenol values of 0.6681–1.7737-mg AG/g/sample, which were quite different among them (*p* < 0.001). A richer concentration was found in the Nariño cultivar. About the antioxidant capacity, according to the DPPH technique, Nariño had the highest value. Based on the FRAP technique, Titicaca and, on the ABTS method, both cultivars had the greatest values (Table 5).

### 2.4. Phenolic Compounds under UHPLC-ESI-Orbitrap MS

The results showed phenolic acid, xanthine, flavonoids and anthocyanin varieties among farms. Contrasting values were observed (*p* < 0.001), highlighting the Nariño cultivar for its apigenin (0.08 ± 0.01) presence and the highest value (42%) of identified elements. Salcedo had an elevated value of caffeic and vanillic acid and Soracá of *p*-coumaric acid and pinocembrin (Table 6).

### 2.5. Phytochemical Multivariate Structure Analysis

**Figure 2:**
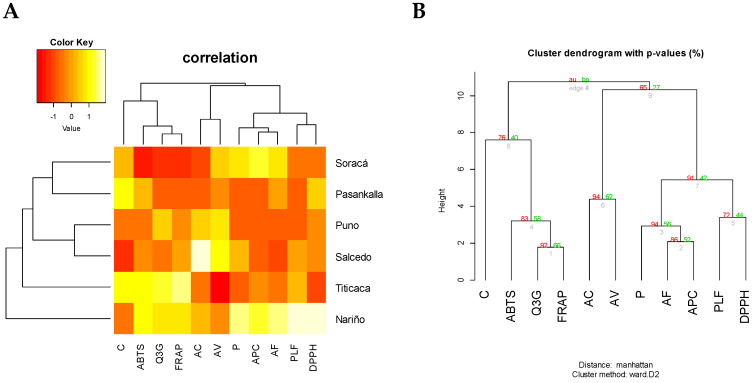
Sample groups according to bioactive compositions from different quinoa seeds. (**A**) Heatmap analysis according to Manhattan distance. (**B**) Boostrap grouping. AU: ap-proximately unbiased. BP: bootstrap probability. C: caffeine, Q3G: quercetin-3-glucoside. AC: caffeic acid; AV: vanillic acid. P: pinocembrin; AF: ferulic acid. APC: *p*-coumaric acid and, PLF: total polyphenols.

The results showed organization in relation to the phenolic components. As noticed in the heatmap, the groupings showed a high diversity in bioactive compounds (Figure 2A). The bootstrap exhibited two main groups: the first includes one xanthine and one anthocyanin and an antioxidant capacity method, while the second combined phenolic acid, a flavonoid, total polyphenols and an antioxidant technique (Figure 2B).

**Figure 3:**
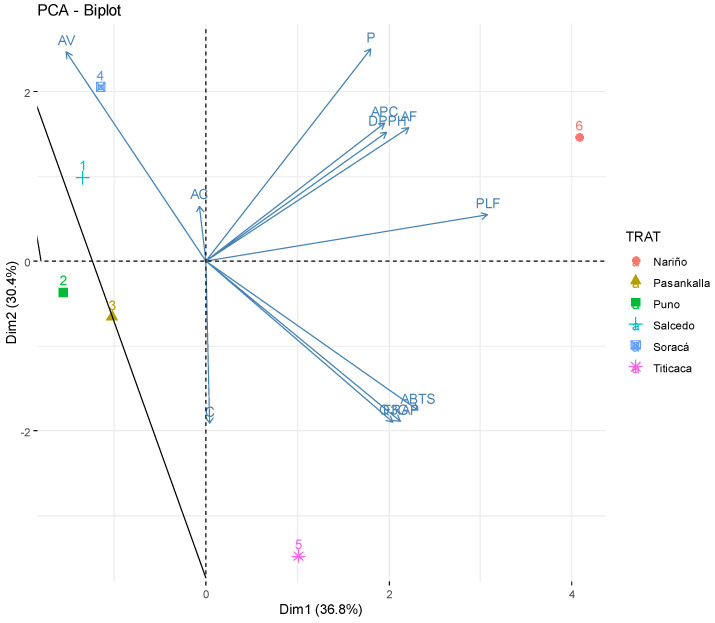
Plot analysis of principal components (PCA) of the bioactive characteristics and antioxidant capacity from six quinoa cultivars in Colombia.

An analysis of the quinoa cultivars’ main components allowed to evaluate the differences in their bioactive composition (Figure 3). The cumulative variance of the two first components was 67.2% (CP); however, when including a third component, the CP variance was 90.8%. It is possible to notice a discrimination of the first two CP from the variance of all six cultivars. The first group’s Nariño cultivar showed the highest amounts of pinocembrin, *p*-coumaric, total polyphenol and high antioxidant capacity with DPPH. Secondly, Titicaca quinoa had greater values of quercetin-3-glucoside, caffeine and antioxidant capacity with ABTS and FRAP. Pasankalla and Soracá presented an observable similarity when adding PC3 as an average cultivar. Finally, Salcedo and Puno were similar because of the presence of some phenolic acids.

## 3. Discussion

The visual aspects of quinoa seeds are determinant on its variety and even compositional differentiation [22]. Over recent years, research evaluating colorimetric coordinates allowed compound identification to boost quinoa cultivars with black, pink, purple and red seeds. Furthermore, it brings about the relationship between tannins, saponins and phytic acid compounds [23,24].

In this sense, protein, lipidic and starchy attributes on grains are fundamental to consider agro-industrial processing or even more, when aiming towards hyper-protein, starch and fat enriched for highly functional quality food [14,25].

To elaborate a structural analysis with the goal to deeply learn about the secondary protein structures of each cultivar, spectrum deconvolution techniques were performed on 1633 cm^−1^ [26]. Deconvolution of the 1016 cm^−1^ band can relate to the crystallinity of a shapeless starch granule [27]. This turned out to be an important fact, because the functional flour does not only depend on the protein and starch concentrations but, also, on its organization and molecular conformation.

The intensity from the amide I band from quinoa flour resulted in 1632 cm^−1^ and 1645 cm^−1^. This condition is relevant to crops’ characterization and differentiation. Nariño and Pasankalla offer a band intensity at 1645 cm^−1^, Puno and Soracá at 1643 cm^−1^, Salcedo at 1632 cm^−1^ and Titicaca at 1633 cm^−1^.

**Figure 4:**
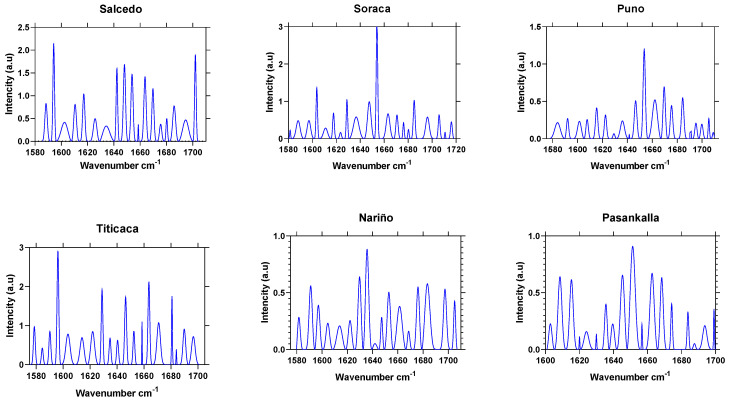
Protein secondary structures of quinoa grains from Colombian cultivars through the deconvolution FTIR technique.

According to Yang et al. (2015) [26], the absorbance peak at the amide I band withstood a deconvolution process to determine the structures like β-sheet (1624–1642 cm^−1^), Random coil (1645–1648 cm^−1^), α-elice (1653–1656 cm^−1^) and β-turns (1667–1694 cm^−1^). All the cultivars displayed contrasting intensity peaks on the secondary structures (Figure 4). Titicaca had a greater β-sheet-1 and -2 proportions, Soracá β-sheet-3, Random coil and α-elice. Titicaca had β-turns-1, while there was β-turns-2 and -3 in Nariño.

In this sense, the discovered results were dramatically different according to other research because of its major manifestation, the peak intensity was found using random coil and α-elice. The results obtained agree with what was stated by Wang, Zhao, and Yuan (2020) [28], who found secondary structures of proteins in the same peak position in the original spectra in this research. Wolkers et al. (1998) [29] explained that such variability could be associated with effects on its production regions because of the edaphoclimatic conditions.

It is also important to outline that secondary structure spectrums could be also used like a fingerprint distinct to each cultivar. Improving the spectroscopy methods could contribute to identify not only the genetic variability but to offer quality control towards adulterated flour [30].

Then, when looking at each cultivar protein fingerprint, it could be possible to assert that the structural differences directly affect the technical and functional capacity to produce gel, foams or emulsions [31].

**Figure 5:**
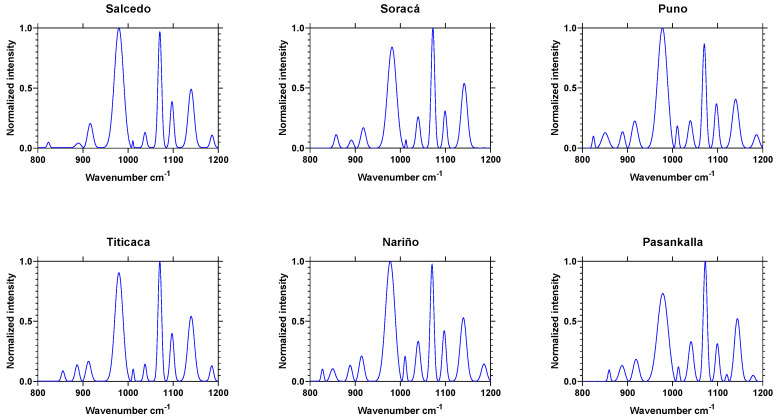
Deconvolution of the starch FTIR band in six Colombian quinoa grain cultivars.

Ratios 1041/1014 and 996/1014 cm^−1^ were used with greater frequency to measure the crystallinity range in starch. Such a fact made it possible to establish that the range 1041/1014 cm^−1^ varied among all six evaluated cultivars (Figure 5). The organized structure of granules indicates that starch changes mostly because of the two conditions determining photoassimilate transports from organs to grains. The first was related to K^+^ movement on apoplast, determining the flow pressure. The second, the invertase acid activity influencing the transforming saccharose in hexoses and restructuring dispose organs for the nutritional quality of seeds [32,33].

Such results are quite important, because to obtain gel or translucent films, is important to use starch with a high crystallinity index, to which the Soracá cultivar is the most suitable (1041/1014 = 4.02). Retrograde phenomenon must be avoided when using flour as the complementing nutritional value. Then, starch with a lower crystallinity index should be used, in which 1.3 and 1.48 indexes from Pasankalla and Nariño are the most appropriate.

In this sense, the high diversity of quinoa makes it possible to find variations in the foliar area of the plants, which is related to the CO_2_ net assimilation and to obtain triose phosphate in the CO_2_ fixation phase, which allows the obtaining of starches for their translocation to sink organs [34,35]. Additionally, Reguera and coworkers found changes in the synthesis of secondary metabolites of three quinoa cultivars and highlighted that these compounds seem to be genotype-specific and could vary significantly under stressful conditions [9].

The polyphenol values vary in all quinoa cultivars and are linked to climatic conditions, agronomical practices and genetic interaction [17]. Li, Lietz & Seal (2021) [36] reported 1.44-mg AG/g of total phenols and antioxidant activity between 8.61 and 522 µm of Trolox/g under ABTS and DPPH. Such results are different from the findings in this paper. The Pasankalla and Salcedo farms reported an antioxidant activity of 5.6- and 3.18-µm Trolox/g under DPPH [37] and a 27.9-µm Trolox/g value with the ABTS technique on a Bolivian farm [38]. Such results are meaningfully high compared to the ones observed here.

This parameter turns to be important to perform a study on how to enrich nutritional models with a low antioxidant capacity, as well as to identify cultivars that might bring about this quality [39]. Likewise, the cultivars with the highest antioxidant capacity were Nariño and Titicaca, because their measurements were highly recognized over other samples during at least two different techniques.

Polyphenols have been recently studied in quinoa, because their diversity is equal or greater than other cereals. Findings of 0.14–0.33 mg/kg of caffeic acid and 0.4- and 0.94-mg/kg vanillic acid were in ten different accessions in Chilean quinoa [18].

Reference Abdelaleem & Elbassiony (2021) [17] reported Egyptian cultivars lacking caffeine but with values of caffeic acid, vanillic acid and apigenin up to 0.49 mg/kg, 30.5 mg/kg and 0.44 mg/kg, accordingly. The quercetin-3-glucosid concentrations observed were considerably low in this research, and the *p*-coumaric acid values reported were similar to those by Tang & Tsao (2017) [40].

The results observed by the heatmap and bootstrap determined the bioactive differences from each evaluated cultivar. It is relevant to mention that many polyphenols are not synthetized on a regular basis in plants, but this could be a result from external conditions such as biotic and abiotic factors [41].

The similarities between quercetin-3-glucoside, FRAP and ABTS happen because of the high capacity from anthocyanins to capture free radicals. In the same way, the concentration resemblance in some phenolic acids occurs because of methylation from a few compounds like caffeic acid, the forefather of vanillic acid [42]. The correlation between ferulic acid and *p*-coumaric acid is a consequence, because they both belong to the hydroxycinnamic group.

It is accurate to mention that this paper reports, for the first time, the presence of pinocembrin and apigenin in quinoa compounds. For other species, its synthesis is attributed to pathogen agents affecting the plant during primary production. Additionally, the influence of edaphoclimatic conditions like salinity, water deficit or very cold weather in amaranth plants was found [43,44].

High contributions on the PCA of ferulic acid and vanillic acid, among other acids, coincide with the ones reported by Antognoni et al. (2021) [45]. They identified that Titicaca has the greatest polyphenol values expressed by these two acids and that its concentration could be greater than 100 mg/kg in quinoa flour.

Carciochi, Manrique & Dimitrov (2014) [46] found a relationship between ferulic acid and vanillic acid concentrations in seeds. Outlining that the two elements are the ones supporting the seed antioxidant activity, in parallel, its content value rises along with the plant growing towards germination.

## 4. Materials and Methods

### 4.1. Materials

We used Salcedo, Puno, Pasankalla, Soracá, Titicaca and Nariño cultivars collected from crops established in the municipalities of Oicatá, Ventaquemada and Tuta in the Department of Boyacá, and others were taken from the seed collection of the “Laboratorio de Biotecnología Vegetal de la Gobernación de Boyacá”. The cultivars were grown in a greenhouse located at Victoria Granja Agroecologica Company, Ventaqumada, Colombia (5°22′47″ N, 73°30′10″ W). The seeds were germinated in 10-kg soil pots. The soil corresponded to an Andosol with the following physicochemical composition: pH 6.1; electrical conductivity 6.3 ds m^−1^; organic matter 8.8%; cations (cmol Kg^−1^) Al^3+^ 1, Ca 36.4, Mg 7.2, K 10.3 and Na 1.1 and micronutrients (mg Kg^−1^) Fe 93.8, Mn 11.8, Cu 9.4, Zn 9.4 and B 1.5. During the study, the irrigation and preventive sanitary management were carried out in order to maintain plant health. The seeds were manually cleaned to remove foreign matter and broken and immature seeds.

The reagents to determine the antioxidant capacity were: free radical DPPH (1,1-diphenyl-2-picryl-hydrazyl), free radical ABTS (2,2′-azinobis(3-ethylobenzothiazoline-6-sulphonate)), iron III chloride (FeCl3); triphenyl tetrazolium chloride (TPTZ), sodium acetate, ascorbic acid, potassium persulfate, Trolox (97%) and ethanol (analytic grade).

The total polyphenol reagents were Folin Ciocalteau, sodium acid carbonate (99%) and gallic acid.

The standard phenolic reference: caffeine (Part N° C8960250G), theobromine (Part N° T4500-25G); theophylline (Part N° T163325G), (±)-catechin (C) (Part N° C1788-500MG), (-)-epigallocatechin gallate (EGCG) (Part N° E4143-50MG), (-)-epicatechin (EC) (Part N° E1753-1G), (-)-epicatechin gallate (ECG) (Part N° E3893-10MG), (-)-epigallocatechin (EGC) (Part N° E3768-5MG), caffeic acid (Part N° C0625), p-coumaric acid (Part N° C9008) and vanillic acid (Part N° 06380590-50MG).

Ferulic acid (Part N° 52229-50MG), rosmarinic acid (Part N° 536954-5G), quercetin (Part N° Q4951-10G) and naringenin (Part N° N5893-1G). Luteolin (Part N° L9283-10MG), kaempferol (Part N° K0133-50MG), ursolic acid (Part N° U6753-100MG), pinocembrin (Part N° P5239) and carnosic acid (Part N° C060910MG).

Apigenin (Part N° A3145-25MG), cyanidin 3-rutinoside (Part N° G36428) and pelargonidin 3-glucoside (Part N° 53489). All of them were bought from Sigma-Aldrich (St. Louis, MI, USA). Quercetin-3-glucoside (Part N° 89230) and Kaempferol-3-glucósido (Part N° 89237) were acquired from Phytolab.

### 4.2. Proximal Characteristics

To determinate the protein content, the Kjeldahl method was used. AOAC 960.52 was multiplied by a conversion factor of 6.25 to measure the total raw protein from the seeds. Fats were defined by the AOAC 922.06 method using Soxhlet (Soxtec 2050). Finally, the total carbohydrates were calculated by the difference (i.e., protein + ash + fat + moisture − 100).

### 4.3. CIEL*a*b* Coordinates Determination

The spectrophotometer used was Konica Minolta (Model CM-5; Tokyo, Japan) to determine the seed color hue from the quinoa cultivars. The results were recorded as L* a* b* values.

### 4.4. Mid-Range Infrared Spectroscopy

IR spectrum was obtained using IRAFFINITY-1S equipment (Shimadzu Corp., Kyoto, Japan). The spectrum was performed by the reflection mode between 400 cm^−1^ and 4000 cm^−1^ and proportional over 32 scans with 4 cm^−1^ and a temperature of 25 °C. In the previous measurements, each cultivar had a blank background spectrum.

A spectrum analysis was made by using OriginPro, 7th version. Measurements were fixed and standardized over a baseline between 0 and 1 (represented in the figures) to show the highest transmittance peaks. Deconvolution was performed on a spectrum using Fourier transform to determine the protein secondary structure changes (band 1600–1700 cm^−1^). Later, a modeling of the Gaussian function spectrum was applied. The starch concentration was studied from its formation on a short-range band between 875 cm^−1^ and 1175 cm^−1^. Lipids were present on the 2800–2900 cm^−1^ range. All samples were analyzed in triplicate.

### 4.5. Polyphenols

From each cultivar, 0.5 g of quinoa flour were mixed. Then, to each sample was added 30 mL of sodium hydroxide 2M and stirring for 10 s with vortex (Fisher Scientific, G560, Bohemia, Waltham, MA, USA). It was left for extraction over 24 h on amber glass at room temperature (25 °C) and mixed in darkness. After that, 5.7 mL of hydrochloride acid were added to the samples upon reaching a pH value of 2. Finally, the samples were centrifugated for 15 min with a rotational speed of 10,000 rpm (HERMLE model Z306, La-Bortechnik GmbH, Wasserburg, Germany), then vacuum-filtrated with Whatman paper (No 1).

Phenolic compounds were determined through the Folin Ciocalteau (FC) method. Triple samples were disposed with 40 µL on a separate 1800 µL with FC reagent. For 15 min, a vortex mix was performed to later rest for 5 min. Afterwards, 1200 µL of sodium acid carbonate (NaHCO_3_) at 7.5% (*m*/*v*) was added and let to settle for 60 min. Absorbance at 765 nm was measured on a V-630 UV-VIS model spectrophotometer (JASCO Inc., Easton, MD, USA). The phenol amounts were measured by sample grams expressed in gallic acid milligrams (mg AG/g).

### 4.6. Antioxidants

#### 4.6.1. FRAP

In a 50-mL falcon tube, 0.4 g of the sample were placed. Then, 10 mL of ethanol were added to begin the extraction process. This was mixed by vortex for 15 s and left to later extract the liquid–solid in the mixer over 30 min at 37 °C. After, the samples were centrifugated (15 min. at 5000 rpm and 4 °C) and later vacuum-filtered with Whatman paper (No 1).

Test tubes with a lid were prepared for 3 different samples: blank, experiment and calibration. For neutral (blank) reagents, the sample had 60 µL of methanol. To begin the reaction, 180 µL of distill water were put in each tube. Then, there was 15 s of vortexing to later add 1800 µL of the FRAP reagent. Next, it was incubated at 37 °C for 30 min.

Finally, 595-nm absorbance was measured on a V-630 UV-VIS model spectrophotometer (JASCO Inc., Easton, MD, USA). The antioxidant activity was expressed with µmol of ascorbic acid/g of the sample (µmol de AA/g).

#### 4.6.2. ABTS

In a 50-mL falcon test tube, 0.4 g from the sample was disposed. To begin the extraction, 10 mL of ethanol were added, vortexed for 15 s and left for solid–liquid extraction over 16 h at 4 °C. Subsequently, the samples were centrifugated over 15 min at 10,000 rpm, then vacuum-filtered with Whatman paper (No 1).

In the test tube, 4 mL of ABTS solution was placed and totally covered with aluminum foil. To begin the process, 135 μL of standard solution were added and then mixed by vortexing for 5 s. Blank reagent consisted of 4 mL of buffer acetate and 135 μL of ethanol. The zero point was mixed with 4.5-mL ABTS solution and 135 μL of ethanol. The test tube was closed and left to react for30 min to finally measure the absorbance at a 729.7-nm wavelength.

#### 4.6.3. DPPH

In a 50-mL falcon test tube, 0.4 g were measured before adding 10 mL of ethanol. Vortexing for 15 s was done and then, the solid–liquid extract was left to settle for 16 h at 4 °C. After such a time, the samples were centrifugated for 15 min at 10,000 rpm, then vacuum-filtered with Whatman paper (No 1).

In a test tube, 3.9 mL of DPPH solution and 100 μL of standard solution were applied to launch a reaction by vortex stirring for 5 s. Blank reagents (control) were carried out with ethanol. The zero point was adjusted by adding 3.9 mL of DPPH solution and 100-μL ethanol. The sample was covered for 30 min to initiate the reaction to later measure the absorbance at 517 nm.

### 4.7. Phenolic Compounds Determination by UHPLC/ESI-Orbitrap MS

The seeds were macerated for four minutes with a blade grinder (Krups; Solingen, Germany). Later, 100 mL of 80% ethanol at 60 °C was added to 5.0 g of quinoa flour. The resultant extract was centrifugated at 10,000 rpm over 10 min. The leftover sample was recovered and stored at 4 °C for later use.

The extract was analyzed on a liquid high-efficiency chromatograph (UHPLC) Dionex Ultimate 3000 (Thermo Scientific, Sunnyvale, CA, USA, EE.UU.). This equipment consists of a binary gradient pump (HP G3400RS) and automatic sample injector (WPS 300TRS) and a thermostatic unit for a TCC 3000 column. The interface of LC-MS was electrospray ionization (ESI). The spectrometer Orbitrap had high mass resolution with ion current detecting system. Chromatography separation was performed by Hypersil GOLD Aq 100 × 2.1 mm, 1.9 µm (Thermo Scientific, Sunnyvale, CA, USA, EE.UU.) column at 30 °C.

Mobile phase A consisted of a 0.2% aqueous solution of ammonium formate. Mobile phase B: 0.2% on acetonitrile ammonia formate. The initial gradient condition was 100%. A moved linearly towards 100% of B in 8 min. Such a phenomena was held for 4 min, then returned to the initial conditions in one minute. The total running time lasted 13 min, plus 3 post-running additional minutes.

Mass spectrometer Orbitrap (Exactive Plus, Thermo Scientific, Sunnyvale, CA, USA, EE.UU.) connected through the electrospray (HESI) interface was handled under a positive capillary voltage of 4.5 kV. Nitrogen gas was used as the dry element. The mass spectrum range was *m*/*z* 60–900. The Orbitrap mass detector was calibrated with certified reference solutions: Ultramark^TM^ 1621 Mass Spec. (AB172435, ABCR GmbH & Co. KG, Karlsruhe, Germany), sodium dodecyl sulfate (SDS) (L4509, Sigma-Aldrich) and sodium taurocholate hydrate (T4009, Sigma-Aldrich).

All compound identification was achieved by using the full scan mode and ion extraction (EIC) corresponding to the [M+H]^+^ of the target compounds. A mass assessment was performed with the accuracy precision of Δ_ppm_ < 0.001 and using a standard mix solution of phenolic compounds. To quantify the samples, the external standard calibration was applied using the results factor (Rf) stablished by the standard solutions analysis under different concentrations.

### 4.8. Statistical Analysis

The values presented are the means ± standard deviation of three replicates. The data were analyzed using the Shapiro–Wilk normality test and Bartlett’s homogeneity test, and the variance analysis (ANOVA) by Tukey’s test was done to observe significant differences (*p* < 0.05). A principal component analysis (PCA) was collected to stablish the relationship of the antioxidant and polyphenol changes among the cultivars using the *Factoextra* and *FactoMinerR* libraries. A bootstrap analysis was elaborated using the *pvclust* function [47]. A double-grouping analysis was performed by Manhattan distance with the *gplots* and *RColorBrewer* libraries [48]. All the analyses were conducted using R version 3.6.1 software.

## 5. Conclusions

A spectroscopy analysis of different quinoa cultivars showed significant variations attributed to their genetic character. This showed that not only external factors but, also, morphological and physiological diversity are influential on the structural configuration of proteins. The same conditions happen to the crystalline/amorphous properties of starch granules.

It is relevant to mention that some alloys like caffeic acid, quercetin 3-glucosid, caffeine and pinocembrin are not very well-identified in quinoa grains; therefore, the influence over its antioxidant activity is still uncertain.

The teamwork between the spectrum deconvolution technique, chromatographic analysis and multivariate analysis are strong tools used for the seed characterization of cultivars from the Colombian Inter-Andean Valley.

## Figures and Tables

**Table 1:** Physical–chemical characteristics in Colombian quinoa grains.

Cultivars	L*	a*	b*	Protein	Carbohydrates	Fat
Titicaca	53.50 ± 0.58 d	4.05 ± 0.08 b	23.84 ± 0.04 b	14.63 ± 0.30 a	49.36 ± 0.70 c	5.7 ± 0.30 c
Salcedo	73.59 ± 0.52 a	1.51 ± 0.03 e	18.14 ± 0.05 e	13.36 ± 0.47 b	59.63 ± 0.47 ab	6.76 ± 0.25 ab
Soracá	69.40 ± 0.90 b	1.93 ± 0.16 d	21.12 ± 0.21 c	14.10 ± 0.26 ab	57.30 ± 0.70 b	6.1 ± 0.1 c
Pasankalla	30.78 ± 0.46 e	10.4 ± 0.24 a	11.92 ± 0.26 f	14. 46 ± 0.14 a	57.30 ± 1.05 b	6.76 ± 0.25 ab
Puno	54.03 ± 0.22 d	1.32 ± 0.19 e	18.99 ± 0.16 d	11.73 ± 0.30 c	61.06 ± 0.51 a	7 ± 0.1 a
Nariño	62.29 ± 0.34 c	3.56 ± 0.04 c	25.59 ± 0.04 a	11.36 ± 0.30 c	62.53 ± 2.05 a	6.26 ± 0.15 bc

Results are expressed as the mean ± standard deviation (*n* = 3). Different letters had significant differences (*p* < 0.05) using Tukey’s test.

**Table 2:** Band intensities distinctive to lipids, proteins and starch.

Cultivars	C-H Stretching (2922 cm^−1^)	C-H Stretching; C-O-C; C-O Bending (1016 cm^−1^)	C=O Stretching (1633 cm^−1^)
Titicaca	0.65 ± 0.02 b	0.0011 ± 0.00006 c	0.47 ± 0.04 b
Salcedo	0.68 ± 0.01 ab	0.0021 ± 0.0001 b	0.34 ± 0.06 c
Soracá	0.69 ± 0.008 ab	0.003 ± 0.0004 a	0.55 ± 0.04 ab
Puno	0.68 ± 0.04 ab	0.0009 ± 0.0004 c	0.30 ± 0.02 c
Pasankalla	0.76 ± 0.04 a	0.0031 ± 0.0001 a	0.61 ± 0.03 a
Nariño	0.68 ± 0.01 ab	0.0021 ± 0.0001 b	0.32 ± 0.005 c

Results are expressed as the mean ± standard deviation (*n* = 3). Different letters had significant differences (*p* < 0.05) using Tukey’s test.

**Table 3:** Secondary protein structures in six Colombian quinoa cultivars.

Cultivars	β-Sheet-1 (1624 cm^−1^)	β-Sheet-2 (1627 cm^−1^)	β-Sheet-3 (1635 cm^−1^)	Random Coil (1648 cm^−1^)	α Elice (1656 cm^−1^)	β-Turns-1 (1667 cm^−1^)	β-Turns-2 (1675 cm^−1^)	β-Turns-3 (1680 cm^−1^)
Titicaca	2.99 ± 0.13 a	2.87 ± 0.07 a	1.09 ± 0.08 c	3.08 ± 0.07 b	1.33 ± 0.07 e	3.53 ± 0.06 a	1.36 ± 0.05 b	0.26 ± 0.05 e
Salcedo	2.14 ± 0.05 b	1.89 ± 0.06 c	3.08 ± 0.07 b	3.35 ± 0.14 ab	3.07 ± 0.07 b	2.14 ± 0.14 b	0.92 ± 0.08 d	0.77 ± 0.05 c
Soracá	0.37 ± 0.04 e	1.49 ± 0.04 d	3.44 ± 0.12 a	3.59 ± 0.14 a	4.97 ± 0.09 a	1.46 ± 0.07 c	0.59 ± 0.01 e	0.33 ± 0.01 de
Puno	0.75 ± 0.04 d	0.17 ± 0.02 e	1.11 ± 0.19 c	1.24 ± 0.1 d	2.56 ± 0.14 c	1.66 ± 0.09 c	1.07 ± 0.04 c	1.31 ± 0.01 b
Pasankalla	0.58 ± 0.02 de	0.08 ± 0.009 e	0.95 ± 0.04 c	2.53 ± 0.1 c	0.14 ± 0.009 f	1.47 ± 0.05 c	0.51 ± 0.03 e	0.5 ± 0.08 d
Nariño	1.1 ± 0.12 c	2.36 ± 0.1 b	3.38 ± 0.13 ab	0.72 ± 0.09 e	1.73 ± 0.07 e	0.57 ± 0.1 d	1.9 ± 0.01 a	3.37 ± 0.09 a

Results are expressed as the mean ± standard deviation (*n* = 3). Different letters had significant differences (*p* < 0.05) using Tukey’s test.

**Table 4:** Band intensities of starch from different Colombian quinoa cultivars.

Cultivars	996 cm^−1^	1014 cm^−1^	1041 cm^−1^	1076 cm^−1^	1099 cm^−1^	1145 cm^−1^	996/1014	1041/1014
Titicaca	0.9 ± 0.01 b	0.11 ± 0.01 c	0.14 ± 0.004 c	0.99 ± 0.007 a	0.39 ± 0.01 ab	0.53 ± 0.01 a	8.23 ± 0.79 b	1.3 ± 0.08 d
Salcedo	0.98 ± 0.01 a	0.06 ± 0.004 d	0.13 ± 0.006 c	0.96 ± 0.02 b	0.38 ± 0.01 ab	0.46 ± 0.01 b	15.28 ± 1.1 a	2.04 ± 0.12 b
Soracá	0.82 ± 0.01 c	0.06 ± 0.01 d	0.26 ± 0.01 b	0.98 ± 0.005 a	0.33 ± 0.03 c	0.53 ± 0.02 a	14 ± 2.1 a	4.02 ± 0.27 a
Puno	0.97 ± 0.01 a	0.16 ± 0.01 b	0.23 ± 0.01 b	0.86 ± 0.005 c	0.34 ± 0.01 bc	0.4 ± 0.01 c	6.11 ± 0.32 bc	1.48 ± 0.16 cd
Pasankalla	0.81 ± 0.01 c	0.17 ± 0.01 b	0.32 ± 0.004 a	0.99 ± 0.002 a	0.31 ± 0.05 c	0.53 ± 0.01 a	4.81 ± 0.34 c	1.91 ± 0.1 bc
Nariño	0.98 ± 0.01 a	0.22 ± 0.02 a	0.34 ± 0.01 a	0.97 ± 0.005 ab	0.41 ± 0.01 a	0.52 ± 0.01 a	4.51 ± 0.53 c	1.58 ± 0.16 bcd

Results are expressed as the mean ± standard deviation (*n* = 3). Different letters had significant differences (*p* < 0.05) using Tukey’s test.

**Table 5:** Phenolic composition and antioxidant activity in quinoa cultivars in Colombia.

Cultivars	Polyphenols (mg AG/g)	ABTS (µmol de T/g)	DPPH (µmol de T /g)	FRAP (µmol de AA/g)
Titicaca	1.0409 ± 0.0202 b	10.8536 ± 0.1317 a	2.3488 ± 0.0177 d	3.2626 ± 0.0585 a
Salcedo	0.9303 ± 0.0103 c	8.2387 ± 0.2718 c	2.7893 ± 0.0627 c	1.9886 ± 0.0643 c
Soracá	0.735 ± 0.0212 d	5.6546 ± 0.1075 d	2.6638 ± 0.0648 c	0.8063 ± 0.0215 f
Puno	0.6782 ± 0.0198 e	7.9468 ± 0.1222 c	2.8127 ± 0.0830 c	1.6469 ± 0.0159 d
Pasankalla	0.6681 ± 0.001 e	9.4534 ± 0.3435 b	3.2587 ± 0.0697 b	1.2685 ± 0.0373 e
Nariño	1.7737 ± 0.009 a	11.0023 ± 0.0857 a	3.8935 ± 0.0454 a	2.6356 ± 0.0212 b

Results are expressed as the mean ± standard deviation (*n* = 3). Different letters had significant differences (*p* < 0.001) using Tukey’s test. AG: gallic acid; T: Trolox; AA: ascorbic acid.

**Table 6:** Phenolic compound contents (mg/Kg) in quinoa seed samples using UHPLC/ESI+-Orbitrap/MS.

Cultivars	Caffeine	Caffeic Acid	Vanillic Acid	*p*-Coumaric Acid	Ferulic Acid	Pinocembrin	Quercetin-3-Glucoside
Salcedo	0.12 ± 0.002 d	2.41 ± 0.02 a	4.02 ± 0.01 a	1.92 ± 0.02 d	5.58 ± 0.1 f	0.04 ± 0.01 bc	0.53 ± 0.09 c
Puno	0.13 ± 0.003 c	1.88 ± 0.01 b	3.66 ± 0.01 b	2.04 ± 0.02 d	6.71 ± 0.09 e	0.01 ± 0.01 c	1.23 ± 0.11 b
Pasankalla	0.15 ± 0.003 a	1.43 ± 0.02 d	3.02 ± 0.01 e	2.6 ± 0.02 c	9.26 ± 0.1 c	0.01 ± 0.01 c	0.37 ± 0.14 cd
Soracá	0.14 ± 0.003 b	1.26 ± 0.01 e	3.49 ± 0.01 c	14 ± 0.2 a	11.83 ± 0.09 b	0.05 ± 0.02 ab	0.1 ± 0.1 d
Titicaca	0.15 ± 0.003 a	1.49 ± 0.01 c	1.94 ± 0.02 f	5.53 ± 0.2 b	7.42 ± 0.1 d	0.01 ± 0.009 c	1.79 ± 0.12 a
Nariño	0.13 ± 0.002 c	1.84 ± 0.01 b	3.07 ± 0.01 d	14.41 ± 0.02 a	14.67 ± 0.1 a	0.07 ± 0.01 a	1.49 ± 0.11 b

Results are expressed as the mean ± standard deviation (*n* = 3). Different letters had significant differences (*p* < 0.001) using Tukey’s test.

## Data Availability

The data generated within this work are open access and available to be shared with interested persons.

## References

[1] Bazile D., Pulvento C., Verniau A., Al-Nusairi M.S., Ba D., Breidy J., Hassan L., Mohammed M.I., Mambetov O., Otambekova M. (2016). Worldwide Evaluations of Quinoa: Preliminary Results from Post International Year of Quinoa FAO Projects in Nine Countries. Front Plant Sci..

[2] García-Parra M., Zurita-Silva A., Stechauner-Rohringer R., Roa-Acosta D., Jacobsen S.-E. (2020). Quinoa (*Chenopodium quinoa* Willd.) and its relationship with agroclimatic characteristics: A Colombian perspective. Chil. J. Agric. Res..

[3] Ruiz K.B., Biondi S., Oses R., Acuña-Rodríguez I.S., Antognoni F., Martinez-Mosqueira E.A., Coulibaly A., Canahua-Murillo A., Pinto M., Zurita-Silva A. (2014). Quinoa biodiversity and sustainability for food security under climate change. A review. Agron. Sustain. Dev..

[4] Pinedo-Taco R., Gómez-Pando L., Julca-Otiniano A. (2020). Sostenibilidad ambiental de la producción de quinua (*Chenopodium quinoa* Willd.) en los valles interandinos del Perú. Cienc. Tecnol. Agropecu..

[5] Bazile D., Martínez E.A., Fuentes F. (2014). Diversity of quinoa in a biogeographical Island: A review of constraints and potential from arid to temperate regions of Chile. Not. Bot. Horti Agrobot..

[6] Curti R., Carmen-Sanahuja M., Vidueiros S., Curti C., Pallaro A., Bertero H. (2019). Oil quality in sea level quinoa as determined by cultivar-specific responses to temperature and radiation conditions. J. Sci. Food Agric..

[7] Li G., Zhu F. (2018). Quinoa starch: Structure, properties, and applications. Carbohydr. Polym..

[8] Bazile D., Bertero H.D., Nieto C. (2014). Estado del Arte de la Quinua en el Mundo 2013.

[9] Reguera M., Conesa C.M., Gil-Gómez A., Haros C.M., Pérez-Casas M.Á., Briones-Labarca V., Bolaños L., Bonilla I., Álvarez R., Pinto K. (2018). The impact of different agroecological conditions on the nutritional composition of quinoa seeds. PeerJ.

[10] García-Parra M., García-Molano J., Deaquiz-Oyola Y. (2019). Physiological performance of quinoa (*Chenopodium quinoa* Willd.) under agricultural climatic conditions in Boyaca, Colombia. Agron. Colomb..

[11] Lesjak J., Calderini D.F. (2017). Increased Night Temperature Negatively Affects Grain Yield, Biomass and Grain Number in Chilean Quinoa. Front. Plant Sci..

[12] Miranda M., Vega-Gálvez A., Martínez E., López J., Marín R., Aranda M., Fuentes F. (2013). Influence of contrasting environments on seed composition of two quinoa genotypes: Nutritional and functional properties. Chil. J. Agric. Res..

[13] García-Salcedo A.J., Torres-Vargas O.L., Ariza-Calderón H. (2018). Physical-chemical characterization of quinoa (*Chenopodium quinoa* Willd.), amaranth (*Amaranthus caudatus*, L.), and chia (*Salvia hispanica*, L.) flours and seeds. Acta Agron..

[14] Roa-Acosta D.F., Bravo-Gómez J.E., García-Parra M.A., Rodríguez-Herrera R., Solanilla-Duque J.F. (2020). Hyper-protein quinoa flour (*Chenopodium Quinoa* Wild): Monitoring and study of structural and rheological properties. LWT-Food Sci. Technol..

[15] Kwil I., Piwowar-Sulej K., Krzywonos M. (2020). Local entrepreneurship in the context of food production: A review. Sustainability.

[16] Rodríguez S.D., López-Fernández M.P., Maldonado S., Buera M.P. (2019). Evidence on the discrimination of quinoa grains with a combination of FT-MIR and FT-NIR spectroscopy. J. Food Sci. Technol..

[17] Abdelaleem M.A., Elbassiony K.R.A. (2021). Evaluation of phytochemicals and antioxidant activity of gamma irradiated quinoa (*Chenopodium quinoa*). Braz. J. Biol..

[18] Carrasco-Sandoval J., Rebolledo P., Peterssen-Fonseca D., Fischer S., Wilckens R., Aranda M., Henríquez-Aedo K. (2020). A fast and selective method to determine phenolic compounds in quinoa (*Chenopodium quinoa* Will) seeds applying ultrasound-assisted extraction and high-performance liquid chromatography. Chem. Pap..

[19] Bazile D., Jacobsen S.-E., Verniau A. (2016). The Global Expansion of Quinoa: Trends and Limits. Front. Plant Sci..

[20] Barth A. (2007). Infrared spectroscopy of proteins. Biochim. Biophys. Acta Bioenerg..

[21] Sadat A., Joye I.J. (2020). Peak fitting applied to fourier transform infrared and raman spectroscopic analysis of proteins. Appl. Sci..

[22] Escribano J., Cabanes J., Jiménez-Atiénzar M., Ibañez-Tremolada M., Gómez-Pando L.R., García-Carmona F., Gandía-Herrero F. (2017). Characterization of betalains, saponins and antioxidant power in differently colored quinoa (*Chenopodium quinoa*) varieties. Food Chem..

[23] Medina W., Skurtys O., Aguilera J.M. (2010). Study on image analysis application for identification Quinoa seeds (*Chenopodium quinoa* Willd) geographical provenance. LWT-Food Sci. Technol..

[24] Abderrahim F., Huanatico E., Segura R., Arribas S., Gonzalez M.C., Condezo-Hoyos L. (2015). Physical features, phenolic compounds, betalains and total antioxidant capacity of coloured quinoa seeds (*Chenopodium quinoa* Willd.) from Peruvian Altiplano. Food Chem..

[25] Li G., Zhu F. (2018). Rheological properties in relation to molecular structure of quinoa starch. Int. J. Biol. Macromol..

[26] Yang H., Yang S., Kong J., Dong A., Yu S. (2015). Obtaining information about protein secondary structures in aqueous solution using Fourier transform IR spectroscopy. Nat. Protoc..

[27] Roa-Acosta D.F., Solanilla-Duque J.F., Agudelo-Laverde L., Villada-Castillo H.S., Tolaba M.P. (2020). Structural and thermal properties of the amaranth starch granule obtained by high-impact wet milling. Int. J. Food Eng..

[28] Wang X., Zhao R., Yuan W. (2020). Composition and secondary structure of proteins isolated from six different quinoa varieties from China. J. Cereal Sci..

[29] Wolkers W.F., Bochicchio A., Selvaggi G., Hoekstra F.A. (1998). Fourier Transform Infrared Microspectroscopy Detects Changes in Protein Secondary Structure Associated with Desiccation Tolerance in Developing Maize Embryos. Plant Physiol..

[30] Zeng H.Y., Cai L.H., Cai X.L., Wang Y.J., Li Y.Q. (2011). Structure characterization of protein fractions from lotus (*Nelumbo nucifera*) seed. J. Mol. Struct..

[31] Sánchez-Mendoza N.A., Ruiz-Ruiz J.C., Dávila G., Jiménez-Martínez C. (2017). Propiedades tecnofuncionales y biológicas de harina, aislado y fracciones proteicas mayoritarias de semillas de *Inga paterno*. CyTAJ Food.

[32] Taiz L., Zeiger E. (2006). Fisiología Vegetal.

[33] Smith M.R., Rao I.M., Merchant A. (2018). Source-Sink Relationships in Crop Plants and Their Influence on Yield Development and Nutritional Quality. Front. Plant Sci..

[34] Hussain M.I., Al-Dakheel A.J., Reigosa M.J. (2018). Genotypic differences in agro-physiological, biochemical and isotopic responses to salinity stress in quinoa (*Chenopodium quinoa* Willd.) plants: Prospects for salinity tolerance and yield stability. Plant Physiol. Biochem..

[35] Cai Z.-Q., Gao Q. (2020). Comparative physiological and biochemical mechanisms of salt tolerance in five contrasting highland quinoa cultivars. BMC Plant Biol..

[36] Li L., Lietz G., Seal C.J. (2021). Phenolic, apparent antioxidant and nutritional composition of quinoa (*Chenopodium quinoa* Willd.) seeds. Int. J. Food Sci. Technol..

[37] Choque-Quispe D., Ligarda-Samanez C.A., Ramos-Pacheco B.S., Leguía-Damiano S., Calla-Florez M., Zamalloa-Puma L.M., Colque-Condeña L. (2021). Phenolic compounds, antioxidant capacity, and protein content of three varieties of germinated quinoa (*Chenopodium quinoa* willd). Ing. Investig..

[38] Paśko P., Bartoń H., Zagrodzki P., Gorinstein S., Fołta M., Zachwieja Z. (2009). Anthocyanins, total polyphenols and antioxidant activity in amaranth and quinoa seeds and sprouts during their growth. Food Chem..

[39] Ballester-Sánchez J., Gil J.V., Haros C.M., Fernández-Espinar M.T. (2019). Effect of Incorporating White, Red or Black Quinoa Flours on Free and Bound Polyphenol Content, Antioxidant Activity and Colour of Bread. Plant Foods Hum. Nutr..

[40] Tang Y., Tsao R. (2017). Phytochemicals in quinoa and amaranth grains and their antioxidant, anti-inflammatory, and potential health beneficial effects: A review. Mol. Nutr. Food Res..

[41] Hinojosa L., González J., Barrios-Masias F., Fuentes F., Murphy K. (2018). Quinoa Abiotic Stress Responses: A Review. Plants.

[42] Šamec D., Karalija E., Šola I., Vujcic V., Salopek-Sondi B. (2021). The Role of Polyphenols in Abiotic Stress Response: The Influence of Molecular Structure. Plants.

[43] Rasul A., Millimouno F.M., Eltayb W.A., Ali M., Li J., Li X. (2013). Pinocembrin: A Novel Natural Compound with Versatile Pharmacological and Biological Activities. BioMed Res. Int..

[44] Sarker U., Oba S. (2018). Salinity stress enhances color parameters, bioactive leaf pigments, vitamins, polyphenols, flavonoids and antioxidant activity in selected Amaranthus leafy vegetables. J. Sci. Food Agric..

[45] Antognoni F., Potente G., Biondi S., Mandrioli R., Marincich L., Ruiz K.B. (2021). Free and conjugated phenolic profiles and antioxidant activity in quinoa seeds and their relationship with genotype and environment. Plants.

[46] Carciochi R.A., Manrique G.D., Dimitrov K. (2014). Changes in phenolic composition and antioxidant activity during germination of quinoa seeds (*Chenopodium quinoa* Willd.). Int. Food Res. J..

[47] Manjarres-Hernández E., Morillo-Coronado A., Ojeda-perez Z., Cárdenas-Chaparro A., Arias-Moreno D. (2021). Characterizacion of the yield components and selection of materials for breeding programs of quinoa (*Chenopodium quinoa* Willd.). Euphytica.

[48] Landinez-Torres A., Panelli S., Picco A.M., Comandatore F., Tosi S., Capelli E. (2019). A meta-barcoding analysis of soil mycobiota of the upper Andean Colombian agro-environment. Sci. Rep..

